# The Function of Naringin in Inducing Secretion of Osteoprotegerin and Inhibiting Formation of Osteoclasts

**DOI:** 10.1155/2016/8981650

**Published:** 2016-01-14

**Authors:** Tong Xu, Lu Wang, You Tao, Yan Ji, Feng Deng, Xiao-Hong Wu

**Affiliations:** ^1^Department of Prosthodontics, The Affiliated Hospital of Stomatology, Chongqing Medical University, No. 426 Songshibei Road, Yubei District, Chongqing 401147, China; ^2^Chongqing Key Laboratory for Oral Diseases and Biomedical Sciences, No. 426 Songshibei Road, Yubei District, Chongqing 401147, China; ^3^Department of Orthodontics, The Affiliated Hospital of Stomatology, Chongqing Medical University, No. 426 Songshibei Road, Yubei District, Chongqing 401147, China

## Abstract

Osteoporosis has become one of the most prevalent and costly diseases in the world. It is a metabolic disease characterized by reduction in bone mass due to an imbalance between bone formation and resorption. Osteoporosis causes fractures, prolongs bone healing, and impedes osseointegration of dental implants. Its pathological features include osteopenia, degradation of bone tissue microstructure, and increase of bone fragility. In traditional Chinese medicine, the herb Rhizoma Drynariae has been commonly used to treat osteoporosis and bone nonunion. However, the precise underlying mechanism is as yet unclear. Osteoprotegerin is a cytokine receptor shown to play an important role in osteoblast differentiation and bone formation. Hence, activators and ligands of osteoprotegerin are promising drug targets and have been the focus of studies on the development of therapeutics against osteoporosis. In the current study, we found that naringin could synergistically enhance the action of 1*α*,25-dihydroxyvitamin D_3_ in promoting the secretion of osteoprotegerin by osteoblasts* in vitro*. In addition, naringin can also influence the generation of osteoclasts and subsequently bone loss during organ culture. In conclusion, this study provides evidence that natural compounds such as naringin have the potential to be used as alternative medicines for the prevention and treatment of osteolysis.

## 1. Introduction

The two main cell types responsible for bone remodeling are osteoblasts and osteoclasts. Molecules that can either increase the proliferation of osteoblasts and/or inhibit the differentiation of osteoclasts can contribute to the formation of new bone.

The main cause of osteoporosis is a dysfunction of osteoblasts and/or hyperfunction of osteoclasts. Therefore, therapeutic strategies are aimed at improving the proliferation and differentiation of osteoblasts, accompanied by either inhibiting osteoclastogenesis or facilitating dysfunction of osteoclasts. Statin, an HMG-CoA (3-hydroxy-3-methylglutaryl coenzyme A) reductase inhibitor, is currently being used clinically for the treatment of osteoporosis. Other drugs include bone resorption inhibitors like bisphosphonates and calcitonin, all of which contribute to the maintenance of bone mass by inhibiting the function of osteoclasts. However, the negative side effects of statin necessitate investigations into alternative treatment strategies. Currently, there is considerable interest in identifying new and better drugs that can not only stimulate bone formation but also suppress the differentiation of osteoclasts.

The hormone VD_3_ is known to play a crucial role in regulating not only calcium homeostasis but also cell growth and differentiation. Osteoblasts are one of the important targets of VD_3_. VD_3_ were shown to stimulate osteoblast activity and promote the release of a variety of osteoclast correlation factors including osteoprotegerin (OPG), RANKL (receptor activator of unclear factor kappa B ligand), M-CSF (macrophage colony-stimulating factor), and IL-6 (interleukin-6). In this way, VD_3_ can control the dynamics of bone formation and resorption [1–5]. In addition, Motoyuki Uchida et al. reported that VD_3_ can induce the expression of matrix metalloproteinases in the MC3T3-E1 osteoblast precursor cell line to trigger and regulate bone resorption [6].

Naringin has osteoinductive properties and was shown to greatly improve the integration of autogenous bone grafts in the skull of rabbits [7]. Additional studies established that the molecular mechanism of function of naringin is similar to statin, namely, complete inhibition of mevalonate and the downstream metabolite of HMG-CoA reductase. On the other hand, Mandadi et al. reported that naringin could affect bone quality, by preserving calcium concentration and increasing antioxidant activity. Additionally, animal studies have shown that naringin possesses antiosteoporotic activity and that it can suppress the formation of osteoclasts [8–10]. To our knowledge, the functional relationship between naringin and osteoclasts has not been reported. In the present study, we investigate the effect of naringin on the differentiation of osteoclasts and the secretion of OPG* in vitro*, specifically in relation to VD_3_.

## 2. Materials and Methods

### 2.1. Animals

#### 2.1.1. Cell Culture

Murine osteoblastic MC3T3-E1 cells were cultured in *α*-MEM (Hyclone, USA) supplemented with 10% fetal bovine serum (FBS, Hyclone, USA). Cells were plated at a density of 3 × 10^5^ cells per 100 mm 24 before treatment. Cells were treated with 10^−7^ M VD_3_ or 10^−7^ M dexamethasone (Dex) (both from Sigma-Aldrich, USA). Naringin (Sigma-Aldrich, USA), at concentrations of 0, 1, 10, and 100 mg/L, was added to the different experimental groups for durations of 72 h, 10 days, 20 days, and 30 days. The medium was changed every three days.

### 2.2. Bone Organ Culture

In this study, we used four-day old SD rats to prepare bone cultures. Approval was obtained from the local institutional review board and that animal care complied with the guidelines of our institution. All experimental procedures were approved by the Medical Experimental Animal Administrative Committee of Chongqing. The calvaria (or skullcap) was dissected out aseptically from rats immediately after they were sacrificed by cervical dislocation. During dissection, the periosteum and dura mater were left intact on the surface of the skull. Each calvaria was trimmed, divided into four pieces (two parietal, one frontal, and one occipital skull bone), and placed into a 24-well tissue culture plate in *α*-MEM supplemented 10% FBS, 20 mM HEPES, 10^−7^ M VD_3_, 10^−7^ M Dex, 100 U/mL penicillin, and 100 *μ*g/mL streptomycin. Cultures were incubated at 37°C in a humidified atmosphere containing 5% CO_2_. Next, we added naringin at concentrations of 0, 1, 10, and 100 mg/L to the test groups after one day of culture, for a duration of 24 h, 3 days, 7 days, and 10 days. The medium was changed every three days. To make comparisons across the different experimental conditions, we used bone tissues extracted from a single litter of mice, unless otherwise indicated by an axis break in the figures.

### 2.3. Reverse Transcription-Polymerase Chain Reaction (RT-PCR)

Total RNA was isolated from MC3T3-E1 cells using TRIZOL reagent (Invitrogen, Carlsbad, CA), according to the manufacturer's instructions. The concentration of RNA was determined by measuring the optical density of the sample at 260 nm. Equal amounts of all RNA samples were then reverse-transcribed. These cDNA samples were then analyzed for the expression of markers of osteogenic differentiation, including OPG, RANKL, M-CSF, and IL-6. The housekeeping gene *β*-actin was used as the internal control for normalizing gene expression level. Using the cDNA as the template, we next performed gene-specific semiquantitative PCR for OPG, RANKL, M-CSF, IL-6, and *β*-actin. Primer sequences and PCR reaction conditions are listed in Table 1. All RT-PCR products were separated and visualized on 1% agarose gel containing 0.5 mg/mL ethidium bromide. To quantify the expression level of the target genes, the DNA bands were first photographed under ultraviolet illumination (Bio-Rad ChemiDoc MP, USA) and then analyzed by densitometry. All PCR reactions generated products in the linear range of amplification, so that band intensity could be directly correlated with the quantity of DNA. Final mRNA levels of all markers were determined after normalizing their densitometry values to *β*-actin (Bio-Rad ChemiDoc MP, USA).

### 2.4. Tartrate-Resistant Acid Phosphatase (TRAP) Staining

Calvarial bone cultures were fixed in 95% ethanol and 5% glacial acetic acid for 10 min, then washed with PBS twice, and stained for TRAP activity using a kit (Sigma-Aldrich, USA), according to the manufacturer's instructions. Following staining at 37°C in a humid and light-protected incubator for 15 min, the cultures were washed with distilled water three times and observed under the microscope. TRAP-positive cells appeared either red or purple. Each stained calvarial bone culture was scanned completely in a raster fashion by transmitted light microscopy (Nikon Eclipse 80i, Japan) at a final magnification of 200x. Finally, the number of TRAP-positive cells in each sample was counted.

### 2.5. ELISA-Based Quantification of Secreted OPG

To detect and estimate OPG levels in the culture medium, we performed ELISA (R&D, USA). Prior to the assay, MC3T3-E1 cells were treated with different concentrations of naringin for 10, 20, and 30 days, while the calvarial bone cultures were treated for 1, 3, 7, and 10 days. The culture medium was collected in Eppendorf (EP) tubes and stored at −80°C until ready for analysis. ELISA was performed according to the manufacturer's instructions. The optical density of immunostained cultures was measured using an ELISA reader.

### 2.6. Measurement of Calcium Concentration in the Bone Culture Medium

To determine the concentration of calcium dissolved from bone slices, the supernatant of calvarial bone cultures treated with different concentrations of naringin was harvested at 1, 3, 7, and 10 days. These samples were collected in EP tubes, stored at −80°C, and diluted 1 : 20 with ultrapure water before assay. The total calcium concentration was determined using an atomic absorption spectrophotometer (AAS) (HITACHI Z-5000, Japan).

### 2.7. Statistical Analyses

Statistical analysis was performed by using the SPSS (SPSS, Chicago, IL, USA) software package. Data from semiquantitative PCR, ELISA, AAS-based Ca^2+^ measurement, and TRAP activity staining are represented as the mean of three independent repetitions of the assay. Krusklal-Walis *H* test was used to determine the statistical significance of differences between the test and control groups. *P* value < 0.05 was considered statistically significant.

## 3. Results

### 3.1. Naringin Induces OPG Expression in Osteoblasts

**Figure 1:**
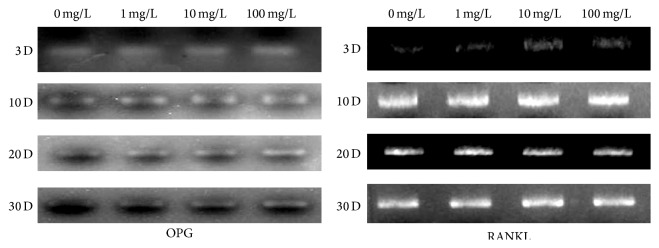
Analysis of OPG and RANKL expression by RT-PCR. Semiquantitative RT-PCR reactions were performed to estimate the expression level of OPG and RANKL in cells cultured under different experimental conditions (0, 1, 10, and 100 mg/L naringin) for 1 to 30 days (D).

To determine the effect of naringin on the expression of osteoprotegerin, we first treated the osteoblast precursor cell line MC3T3-E1, with different concentrations of naringin for different periods of time. Next, we extracted total RNA from control and treated cultures at different time points and measured the mRNA levels of OPG, RANKL, M-CSF, and IL-6 using semiquantitative RT-PCR. Compared with the control group, we found a significant increase in OPG mRNA expression in naringin-exposed cultures in a time- and dose-dependent manner (Figure 1). In contrast, the expression of RANKL, M-CSF, and IL-6 remained the same in control and experimental groups.

### 3.2. Naringin Inhibits the Generation of Osteoclasts in a Time- and Concentration-Dependent Manner

**Figure 2:**
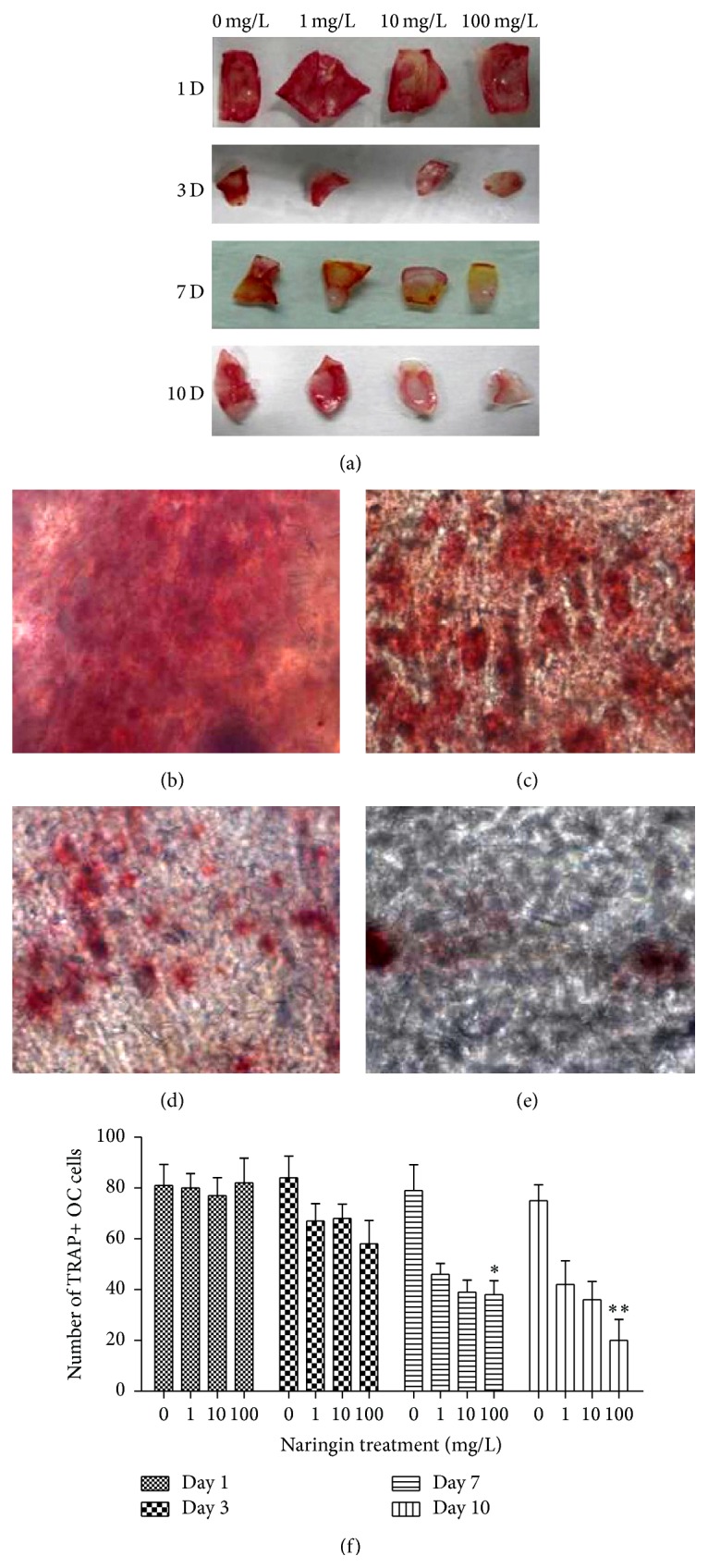
Effect of naringin on the number of TRAP+ OC cells in calvarial bone cultures. (a) Macroscopic view of TRAP staining in calvarial bone cultures in different experimental conditions (0, 1, 10, and 100 mg/L naringin), after 1, 3, 7, and 10 days (D)* in vitro*. (b, c, d, and e) Microscopic view of TRAP staining in calvarial bone cultures in different experimental conditions (0, 1, 10, and 100 mg/L naringin), after 10 days (D)* in vitro*. (f) Quantification of the number of TRAP+ OC cells in bone cultures exposed to 0, 1, 10, and 100 mg/L naringin for 1, 3, 7, and 10 days* in vitro*. Note the significant reduction in TRAP+ cells at 7 days (^*∗*^ *P* < 0.05) and 10 days (^*∗∗*^ *P* < 0.01).

We examined the effects of naringin on the composition of calvarial cultures by first staining for TRAP activity and identified a large and significant reduction in the number of TRAP+ osteoclasts (OC) after at least 3 days of exposure (Figure 2).

After 24 h of culture, we found no difference in the number of TRAP+ OC cells between control and naringin-treated groups. However, after calvarial bones were cultured for 3 days, the number of TRAP+ OC cells generated in culture was reduced by 32%, 19%, and 26% in response to 100 mg/L, 10 mg/L, and 1 mg/L naringin, respectively, when compared with control cultures. After 7 days* in vitro*, we found 67%, 53%, and 44% significant reduction in the quantity of TRAP+ OC cells in response to 100 mg/L, 10 mg/L, and 1 mg/L naringin, respectively (*P* < 0.05). Finally, after 10 days* in vitro*, we found 74%, 52%, and 41% significant reduction in the number of TRAP+ OC cells in response to 100 mg/L, 10 mg/L, and 1 mg/L naringin, respectively (*P* < 0.01).

### 3.3. Naringin Induces Upregulation of OPG in MC3T3-E1 Cells and Calvarial Bone Cultures

To establish whether naringin could influence the secretion of OPG from osteoblast precursors, we assayed for OPG protein levels in the culture supernatant using ELISA. Under control conditions, we detected basal levels of OPG in the culture media of both MC3T3-E1 cells and calvarial bone cultures. However, on exposure to high concentrations of naringin (100 mg/L), these levels were significantly upregulated.

### 3.4. Naringin Exposure Alters Concentration of Dissolved Calcium in Bone Culture Media

**Figure 3:**
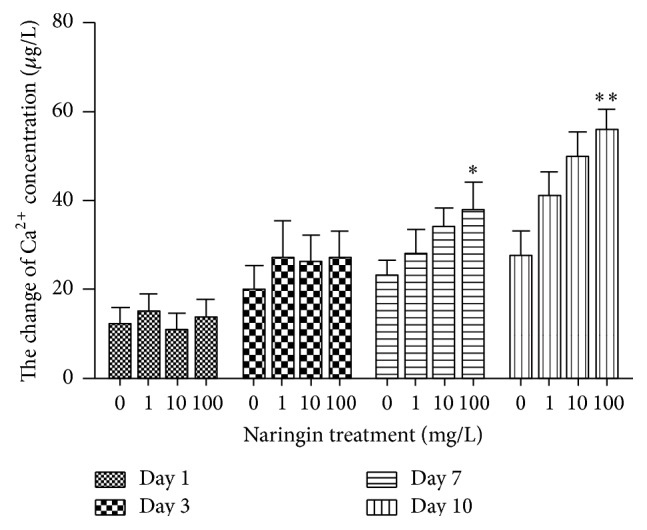
Analysis of calcium released into bone culture media. The estimated concentration of dissolved calcium in the supernatant of calvarial bone cultures under different concentrations of naringin (0, 1, 10, and 100 mg/L) for 1, 3, 7 (^*∗*^ *P* < 0.05), and 10 days (^*∗∗*^ *P* < 0.01)* in vitro* is shown here.

To estimate the effect of naringin on bone formation versus resorption, we quantified the concentration of dissolved calcium ions in culture supernatant under different experimental conditions. The results of AAS- based calcium estimation are shown in Figure 3. We found that after 1 day of exposure to naringin, calcium concentrations were 12 *μ*g/L, 15 *μ*g/L, 10 *μ*g/L, and 13 *μ*g/L in cultures treated with 100 mg/L, 10 mg/L, 1 mg/L, and 0 mg/L naringin, respectively. After 3 days in culture, the concentrations were 27 *μ*g/L, 26 *μ*g/L, 27 *μ*g/L, and 20 *μ*g/L in response to 100 mg/L, 10 mg/L, 1 mg/L, and 0 mg/L naringin, respectively. On day 7, we detected significant differences in calcium levels, namely, 38 *μ*g/L, 34 *μ*g/L, 28 *μ*g/L, and 23 *μ*g/L in response to exposure to 100 mg/L, 10 mg/L, 1 mg/L, and 0 mg/L naringin, respectively (*P* < 0.05). Finally, after 10 days* in vitro*, we again found significant differences in calcium concentrations, specifically, 56 *μ*g/L, 50 *μ*g/L, 41 *μ*g/L, and 28 *μ*g/L in cultures treated with 100 mg/L, 10 mg/L, 1 mg/L, and 0 mg/L naringin, respectively (*P* < 0.01).

## 4. Discussion

Osteoporosis is a major, commonly occurring health concern that can result in fractures. Chinese herbal medicines have been used to treat this condition. The compound naringin has been identified as an active component of Rhizoma Drynariae. In recent years, an increasing number of studies have shown that naringin has antioxidant and free radical scavenging properties and that it can protect cells against free oxygen free radical-stimulated K^+^ permeability. In addition, it can induce BMP-2 expression in osteoblasts and enhance osteogenic differentiation and new bone formation [11–15].

Osteoprotegerin was recently described as a member of the TNF receptor superfamily and its cognate ligand, RANKL, has been identified as an important regulator of bone metabolism that mediates paracrine signaling between osteoblasts and osteoclasts [16]. OPG is produced by many different cell types, including bone marrow stromal cells and osteoblasts. It is known to specifically block the fusion and differentiation of osteoclast precursors, and not their proliferation, by binding to RANKL [17]. The physiological function of OPG in regulating bone mass was revealed by studying transgenic mice overexpressing OPG that showed increased skeletal radio density. Significant levels of osteopetrosis and increased bone density observed in these mice were attributed to a decrease in the number of mature osteoclasts. Together with the observation that OPG knockout results in increased bone resorption and skeletal pathologies reminiscent of osteoporosis, the above data establish OPG as a negative regulator of bone resorption* in vivo*. RANKL is a type II homotrimeric transmembrane protein expressed by cells of the osteoblastic lineage, T lymphocytes, and synoviocytes. It can promote the formation, differentiation, and activation of osteoclasts and lead to enhanced bone resorption. Recent reports have shown that the ratio of OPG to RANKL is an important determinant of bone metabolism and the physiological dynamics between osteoblasts and osteoclasts [18]. In the current study, we have shown that naringin can inhibit the differentiation of osteoclasts by inducing an increase in the OPG/RANKL ratio, resulting from the upregulation of OPG expression rather than downregulation of RANKL.

The hormone VD_3_ can induce osteoblasts to release a variety of osteoclast correlation factors such as OPG, RANKL, M-CSF, and IL-6. Interestingly, M-CSF and IL-6 are also secreted by osteoblasts and multipotent stem cells and appear to be essential for the proliferation and differentiation of osteoclast progenitors [18–20]. Results from the RT-PCR and ELISA assays presented in this report suggest that exposure to naringin could induce an increase in the activity of VD_3_ in osteoblast precursor cells leading to a dose-dependent increase in the expression and secretion of the osteoblast differentiation marker OPG.

To the best of my knowledge, this study is the first to investigate the relationship between osteogenic differentiation and naringin activity during bone remodeling* in vitro*. In contrast to osteoblast precursor cell lines, calvarial bone cultures are composed of all types of cells of the osteoblastic lineage, as well as the bone matrix found inside osseous tissue* in vivo*. Hence, it is more representative of physiological bone growth and metabolism. In comparison to* in vivo* experiments, bone organ cultures can be established in a fast, simple, and economical manner and allow an accurate control of the dose and duration of the compounds being tested. They also have the added advantage of eliminating variability arising from the extracellular matrix, thus improving the reproducibility of the results obtained. The calvarial bone culture system utilized in this study can be used not only to observe bone formation but also to test the effect of drugs and orthopedic materials on the dynamics of bone tissue growth and function. Therefore, bone organ cultures are a good model to study autocrine and paracrine signaling during bone metabolism [21]. In this study, we show that the highest concentrations of naringin (100 mg/L) induced that largest decrease in the number of TRAP+ osteoclasts (74% reduction). These observations are consistent with results from a previous study where TRAP+ OC cells were found to disappear from the endocranial surface of mouse calvarial bones in the presence of naringin. Other studies have shown that naringin can suppress bone resorption induced by positive regulators for osteoclastogenesis including LPS nitric oxide (NO), NO synthase (iNOS), TNF-alpha, inducible cyclooxygenase (COX-2), interleukin-6 (IL-6), RANKL-induced NF-kappa B, and ERK activation [22–26]. Moreover, the reduction in osteoclast differentiation in this study is also consistent with the increase in soluble calcium levels in the organ culture supernatant in the presence of naringin. Altogether, our results suggest that naringin can not only improve bone quality but also reduce the formation or enhance the apoptosis of osteoclasts.

## 5. Conclusion

We show that naringin can increase the activity of VD_3_ in cultured bone cells and induce them to secrete osteoprotegerin, leading in turn to a reduction in the number of osteoclasts and prevention of bone loss. This corroborates previous evidence of the role of naringin in suppressing bone resorption. We therefore conclude that naringin could be highly beneficial in treating osteoporosis, where it could facilitate bone formation by stimulating increased expression of OPG.

## Figures and Tables

**Table 1:** Primer sequences for RT-PCR.

Gene	Primer sequence (forward/reverse)	*T* (annealing)	Cycles
OPG	5′-TCCTGGCACCTACCTAAAACAGCA-3′ 5′-CTACACTCTCGGCATTCACTTTGG-3′	57	35

RANKL	5′-ATGATGGAAGGCTCATGGTTG-3′ 5′-TGTTGGCGTACAGGTAATAGAA-3′	60	35

M-CSF	5′-AAGCAGTAACTGAGCAACGGG-3′ 5′-CCCATATTGCGACACCGAA-3′	59	30

IL-6	5′-GAAATGAGAAAAGAGTTGTGC-3′ 5′-ATTGGAAATTGGGGTAGGAAG-3′	56	35

*β*-actin	5′-TGGAATCCTGTGGCATCCATGAAAC-3′ 5′-TAAAACGCAGCTCAGTAACAGTCCG-3′	61	30
